# Analysis of Tonsillar NK Cell Markers in Pediatric Epstein–Barr Virus (EBV) Asymptomatic Infection and EBV-Associated Hodgkin Lymphoma

**DOI:** 10.3390/v18060667

**Published:** 2026-06-12

**Authors:** Natalia M. Ferressini Gerpe, María E. Amarillo, Oscar Jimenez, Agustina Moyano, María S. Caldirola, María I. Gaillard, Elena De Matteo, Paola Chabay

**Affiliations:** 1 Molecular Biology Laboratory, Pathology Division, Multidisciplinary Institute for Investigation in Pediatric Pathologies (IMIPP-CONICET-GCBA), Ricardo Gutierrez Children’s Hospital, Gallo 1330, Ciudad Autónoma de Buenos Aires C1425EFD, Argentina; nferressini@gmail.com (N.M.F.G.); maria.eugenia.amarillo41@gmail.com (M.E.A.); agus.moyano@hotmail.com (A.M.); elenadematteo@gmail.com (E.D.M.); 2 Immunology Division, Ricardo Gutierrez Children’s Hospital, Gallo 1330, Ciudad Autónoma de Buenos Aires C1425EFD, Argentina; mariasoledadcaldirola@gmail.com (M.S.C.);; 3 Pathology Division, Ricardo Gutierrez Children’s Hospital, Gallo 1330, Ciudad Autónoma de Buenos Aires C1425EFD, Argentina

**Keywords:** EBV, NK cells, IFN γ, tonsil, Hodgkin lymphoma, pediatric

## Abstract

In Argentina, a high incidence of EBV-associated lymphomas was demonstrated in young children. Natural killer (NK) cells, particularly, IFN-γ-producing CD56bright NK cells, have been reported to play a key role in asymptomatic EBV infection in children, restricting viral-mediated transformation. In order to analyze NK cell characteristics in children with primary and persistent EBV infection, along with EBV+ Hodgkin lymphoma (HL) from Argentina, a cohort of EBV-infected pediatric patients was analyzed. A scarcity of CD56+ cells, as an indirect marker of NK cells, across all tonsillar samples and pediatric classical Hodgkin lymphoma cases was observed, with no significant differences according to EBV status. In primary infection, CD56+ cells showed a positive correlation with IFNγ+ cells, suggesting a role in early antiviral responses. Flow cytometry revealed an increased proportion of CD56bright NK cells in EBV-infected children, particularly in cases expressing latency II/III antigens. A significantly higher IFN-γ production was observed in CD56bright cells in children with primary infection compared with healthy carriers, along with an inverse correlation between IFN-γ production and CD56bright cells in healthy carriers. These findings suggest that NK cells may contribute to immune control predominantly during primary infection, whereas their role appears limited in healthy carriers and in EBV-associated Hodgkin lymphoma.

## 1. Introduction

Epstein–Barr virus (EBV) usually behaves as a harmless passenger, since it infects more than 90% of the worldwide population and resides for life in B cells, its target cells, thanks to its sophisticated persistence, maintained in a delicate balance with the host immune response [1]. Disruption of this tightly regulated balance could result in an EBV-associated B-cell lymphoma, owing to the virus’s oncogenic potential demonstrated in vitro, leading to malignancies such as Burkitt lymphoma (BL), Hodgkin Lymphoma (HL) and Diffuse Large B-Cell Lymphoma (DLBCL) [2]. Classical B-cell growth transformation is achieved through the action of nine latent antigens, along with the expression of lytic cycle antigens [3]. The latency III pattern includes the expression of all EBNA and LMP proteins, as well as the EBER and BART transcripts, while in Latency II, the expression of EBNAs is downregulated. Latency I is characterized by the presence of both the EBNA1 protein and non-coding viral transcripts, whereas in Latency 0 the virus persists silently, possibly expressing only EBERs and other non-coding transcripts as a circular genome [4].

Many of these viral proteins elicit antibody and cell-mediated immune responses as well, to control primary infection and to limit reactivation of the persistent infection from its latent reservoir [5]. There are two very different scenarios concerning immune response in primary infected (PI) individuals. In adults, CD8+ T cells are greatly expanded, and this expansion results in the classic symptomatic infection known as infectious mononucleosis (IM). In contrast, in children the infection is mostly asymptomatic, no disruption within the T-cell compartment is observed. Furthermore, it seems that natural killer (NK) cells play a key role in preventing the development of IM symptoms in children [6]. In fact, in a mouse model, the depletion of NK cells enhances IM symptoms and promotes EBV-associated tumorigenesis, mainly because of a loss of immune control over lytic EBV infection [7].

NK cells are members of the innate immune system. Their activity is controlled by self-recognition molecules, and they elicit anti-tumoral and antiviral responses in the missing-self scenario. These functions are accomplished due to their cytotoxic capacity against tumoral or infected cells on one hand, and, on the other, the production of various cytokines, such as IFNγ [8]. In tumors, the interactions between NK cells and other cells within the tumor microenvironment (TME) are crucial determinants of the immune response against cancer. However, NK cells are insufficient in number in various TMEs. Indeed, a substantial proportion of human tumor lesions lack NK cells, and, in contrast, they show a dense infiltration of macrophages that are considered to possess immunosuppressive properties [9]. In Hodgkin lymphoma (HL), the immunosuppressive nature of the TME specifically inhibits the proliferation and activity of NK cells, which contributes to tumor immune-escape mechanisms [10]. The contribution of NK cells to the TME in pediatric HL remains unknown.

The influence of EBV on microenvironment composition has been largely portrayed in adult Hodgkin lymphoma (HL). In this scenario, the coexistence of a functional Th1-cell infiltrate with Treg cells was described, along with a marked number of activated CD8+ T cells as well as NK cells [11]. In adult patients with IM, tonsillar CD56+ NK cells were the least prevalent cell subset in the tissue microenvironment, but a trend toward an inverse correlation between the numbers of EBV+CD20+ and CD56+ NK cells was described, reinforcing the role of NK cells in the control of primary EBV infection [12]. Remarkably, in pediatric tonsils, the mean CD56+ NK cell count was lower around EBV LMP1+ cells, in particular in the subepithelial region. In addition, GzB+ cells were more numerous surrounding cells expressing viral LMP1, suggesting that NK cells were not involved in cytotoxic activity in this specific tonsillar region [13]. These findings prompted us to further characterize the involvement of CD56+ NK cells in the microenvironment immune response during EBV infection and EBV-associated HL in children from Argentina. This region shows a high incidence of pediatric EBV-associated lymphomas [14], which could be related to an imbalance in the control of primary EBV infection and persistence.

## 2. Materials and Methods

### 2.1. Patients and Samples

Formalin-fixed paraffin-embedded (FFPE) and fresh tonsil tissue samples were collected from 74 children aged between 1 and 15 years (median: 5 years) undergoing tonsillectomy due to non-reactive hyperplasia at the Otorhinolaryngology Division, Ricardo Gutierrez Children’s Hospital (Buenos Aires, Argentina). Tonsillar hyperplasia was diagnosed according to international routine protocols for recurrent chronic inflammation. Tonsils were not acutely swollen at the time of removal. In addition, samples from 39 patients with classical HL (cHL) were collected retrospectively, based on the availability of sufficient material, from the archives at the Pathology Division, Ricardo Gutierrez Children’s Hospital in Buenos Aires, Argentina. The age range was 2–18 years (median: 9.5 years). The samples were obtained at diagnosis before treatment, which followed the Grupo Argentino de Tratamiento de la Leucemia Aguda (GATLA) protocol. 

Fresh tissue samples from 41 patients undergoing tonsillectomy were mechanically disrupted in PBS buffer and filtered using sterile gauze; then a density gradient separation method using Ficoll (Sigma Aldrich, Santa Clara, CA, USA) was performed to isolate tonsillar mononuclear cells (TMCs). TMCs were preserved in liquid nitrogen. This analysis enlarges upon a previously published characterization of EBV infection [15].

Institutional guidelines regarding human experimentation were followed, according to the Helsinki Declaration of 1975. The protocol was approved by the Ethical Committee of our hospital, and written informed assent and/or consent were obtained from all patients or patients’ parents, depending on age.

### 2.2. Serological Profile

Five mL of blood samples from each patient, taken at the same time as the tonsillectomies, were incubated at 37 °C for 30 min and then centrifuged for 10 min at 3500 rpm. Serum was collected and stored for indirect immunofluorescence (IF) assays, as previously reported [14]. Primary infected (PI) patients were defined by the presence of IgM and IgG antibodies to VCA (viral capsid antigen); healthy carriers (HC) by the presence of IgG antibodies to VCA and EBNA1; whereas viral reactivation (R) was identified by IgG anti-VCA, anti-EBNA1 and anti-EA (early antigen) antibodies [16]. The serological classification is detailed in Supplementary Table S1.

### 2.3. Immunohistochemistry (IHC) for Viral Antigen Expression

To explore the expression of EBV latent and lytic antigens, IHC for latent antigens LMP1 (CS1-4 pool of clones, catalog 0897, Dako, Glostrup, Denmark) and EBNA2 (R3 clone, ab252828, Abcam, Cambridge, UK), as well as the BMRF1 (G3-E31 clone, ab49668, Abcam) early lytic antigen, was performed on FFPE tonsil samples, as described [15]. The positive cell count in the whole tonsil section was assessed for each protein, and the results were expressed as positive cells per unit area (cells/cm^2^). To detect the presence of EBERs, in situ hybridization with the ViewRNA ISH Tissue 1-Plex Assay and specific probes (catalog 19931, Affymetrix, Brea, CA, USA) was performed according to the manufacturer’s instructions.

### 2.4. IHC Immune Response Markers

To investigate the relationship between CD56 as an NK cell marker and other immune cell markers with immunosuppressive properties, IHC with specific antibodies was performed on FFPE samples: CD56 (clone MRQ-42, catalog 156R-98, Cell Marque, Rocklin, CA, USA), IFNγ (clone 466, ab218426, Abcam), Granzyme B (GzB) (clone GB7, catalog MCA2118, BioRad, Hercules, CA, USA), CD68 (clone KP1, catalog 05278252001, Roche Ventana, Tucson, AZ, USA), CD4 (clone SP35, catalog 05552737001, Roche Ventana), Foxp3 (clone 236A/E7, ab20034 Abcam), CD163 (clone MRQ-26, catalog 05973929001, Roche Ventana), IL10 (polyclonal, ab34843, Abcam), TGFβ (polyclonal, ab9758, Abcam) and PDL1 (clone ABM4E54, ab210931, Abcam), as previously reported [16,17]. The number of labeled cells was determined using the image analysis free software Image J 1.53. The cells were counted optically without the use of a plug-in. The results were expressed as positive cells per unit area (cells+/mm^2^).

### 2.5. Flow Cytometry (FC)

The TMCs preserved in liquid nitrogen were rapidly thawed at 37°, washed and diluted to reach a concentration of 10 million cells per ml. A volume of 100 ul (106 cells) per tube was stained with specific antibodies. The CD56bright and CD56dim cell populations were assessed using antibodies CD3-APC (clone SK7, catalog 344811, CD56-PECy7 (clone HCD56, catalog 318317, Biolegend, San Diego, CA, USA) and CD16-FITC (clone 3G8, catalog 302005, Biolegend). Cells were acquired on a FACSCanto II flow cytometer (BD) and analyzed using FlowJo v7.4 software (BD Life Sciences, Ashland, OR, USA).

To determine NK cells’ capacity to produce IFNγ, 10^6^ TMCs from a subgroup of patients were stimulated overnight in the absence or in the presence of rIL-12 (10 ng/mL), rIL-15 (2 ng/mL) from (PrepoTech, Cranbury, NJ, USA) and rIL-18 (10 ng/mL). During the last 4 h, Golgi-Stop^®^ (BD) was added to the cultures. Afterwards, cells were fixed and permeabilized using Citofix/Citoperm (BD) and stained with anti-IFNγ-PE (clone B27, catalog 554701, BD), anti-CD56-APC (NCAM-1, clone 159, catalog 555518, BD) and anti-CD3-PECy7 (clone UCHT1, catalog 6607100, Beckman Coulter, Brea, CA, USA). Cells were acquired on a FACSCanto II flow cytometer (BD) and analyzed using FlowJo software (BD Life Sciences, Ashland, OR, USA) IFN-γ production was assessed by FC. In the IFNγ assay, positive cells’ mean fluorescent intensity (MFI) was normalized to the unstimulated control, and represented as ΔMFI. Positivity gates were adjusted using a fluorescent minus one (FMO) control.

### 2.6. Statistical Analysis

The data was analyzed using GraphPad Prism 5 software. A normality test was performed using the Shapiro-Wilks test. Comparisons between groups were assessed by the *t*-test and ANOVA, or the Mann–Whitney and Kruskal–Wallis test, according to the normality test results, and correlations were tested using the Pearson or Spearman tests, based on the normality test results. Categorical variables were analyzed with the Fisher’s exact test or the chi-square test. Outliers were defined using a robust test to compare the data’s median absolute deviation (Mad) in Excel^®^ and were excluded from the analysis. Dunn and Tukey comparisons between all columns as a post-test for our non-parametric and parametric multiple group comparisons, respectively, were performed. All tests were two-tailed, and *p* < 0.05 was considered statistically significant.

## 3. Results

### 3.1. EBV Characterization

In a previous study of a cohort of pediatric EBV carriers, our group described a decrease in the number of CD56-positive cells around EBV-infected cells [13]. In light of these results, our purpose was to characterize CD56+ NK cells in the context of different EBV infection statuses and in EBV-associated pediatric cHL. Among the latter group, EBER and LMP1 expression was detected in 30 of 39 cases, indicating that 77% of pediatric cHL cases were EBV+, and all EBV+ cases displayed a latency II pattern (Supplementary Table S2). Regarding children with different EBV infection statuses, 23 patients were primary infected (PI), 37 were healthy carriers (HC), 10 were children undergoing viral reactivation (R) and four were non-infected (NI), based on IF assays of peripheral blood (Supplementary Table S1). The expression of latency antigens allowed us to classify the patients according to the expression of latency proteins, even though only a few cells express latency antigens in children with asymptomatic infection [15]. Thirty-one tonsillar sections expressed latency 0/I antigens (EBERs+/LMP1−/EBNA2−), 19 expressed latency II antigens (EBERs+/LMP1+/EBNA2−), four expressed latency IIb antigens (EBERs+/LMP1−/EBNA2+) and 16 showed latency III antigens (EBERs+/LMP1+/EBNA2+), based on IHC of FFPE tonsil sections, combined with a positive EBV serology result. Sections from children displaying latency IIb antigens were grouped as latency III for further analysis. In addition, 18 cases also exhibited cells expressing the lytic cycle BMRF1 antigen.

### 3.2. CD56 Expression in Relation to Immune Cell Markers

**Figure 1:**
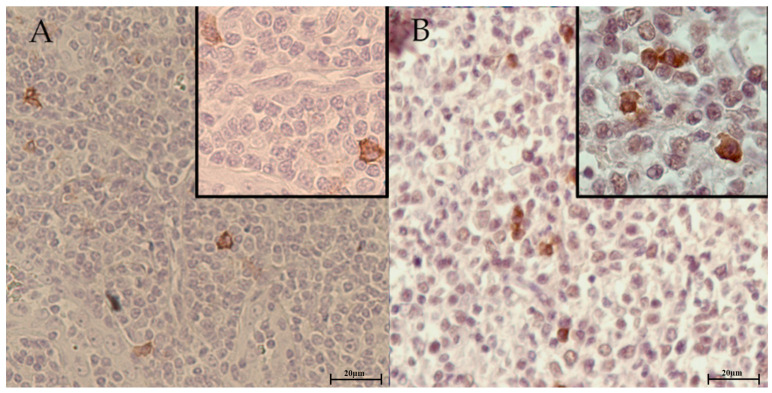
Immunohistochemistry (IHC) on formalin-fixed paraffin-embedded (FFPE) tonsil tissue samples from pediatric patients. (**A**) CD56 positive stain at the cell membrane. (**B**) IFNγ positive cytoplasmic stain. Original magnification ×400, insets ×1000.

**Figure 2:**
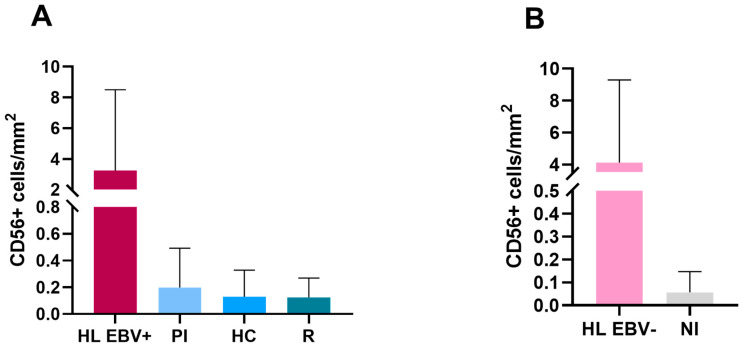
Analysis of the CD56+ cell count from the IHC comparing the three EBV+ infectious status: primary infected (PI), healthy carriers (HC) and reactivation (R) with EBV+ HL (**A**); and non-infected (NI) with EBV− HL (**B**).

IHC staining was performed with specific antibodies against CD56 as an indirect marker of NK cells, and CD56 staining was analyzed in relation to GzB and IFNγ as indicators of cytotoxic activity and cytokine production, respectively (Figure 1A,B). In addition, CD56 expression was analyzed in relation to CD4 and Foxp3, as markers of regulatory T cells; CD68 and CD163 as markers of M1- and M2-like macrophage polarization, respectively; and IL10, TGFβ, and PDL1 as markers of an immunosuppressive microenvironment. Low numbers of CD56+ cells were observed in pediatric cHL, as well as in tonsils from children with different EBV infection statuses. No differences between EBV+ and EBV− cases were observed in the mean CD56 cell counts (*p* > 0.05, Mann–Whitney test) for both the malignant (HL) and non-malignant scenarios (tonsil tissue). In addition, when CD56+ cell counts were compared among EBV-infected children classified as PI, HC, R and NI, no statistical difference was found (*p* > 0.05, Mann–Whitney and Kruskal- Wallis tests). Even though CD56 expression was higher in pediatric cHL than in tonsils across different EBV infection statuses, no statistical differences were observed (*p* > 0.05, Kruskal–Wallis test) (Figure 2).

**Figure 3:**
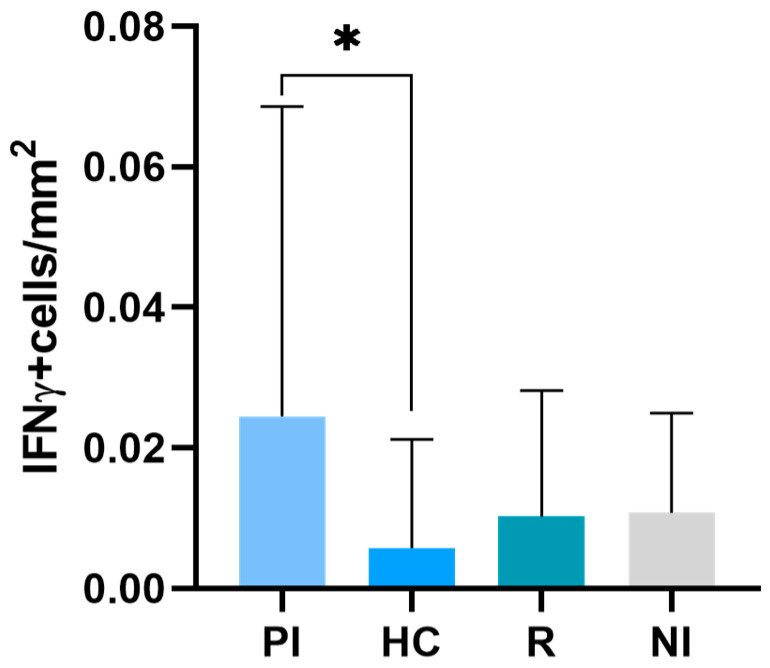
Analysis of IFNγ+ cell counts from IHC comparing the four EBV infectious statuses: primary infected (PI), healthy carriers (HC), reactivation (R) and non-infected (NI). * *p* < 0.05.

After this analysis, correlations between CD56+ cells and the analyzed immune microenvironment cell markers were evaluated as markers of immune cell activation in relation to CD56+ cells. In the entire cohort of pediatric cHL, CD56+ cells showed a statistically significant positive correlation with CD4+ T cells (*p* = 0.034, r = 0.355; Spearman correlation test), and this association was observed particularly in EBV+ cases (*p* = 0.012, r = 0.466, Spearman correlation test). In contrast, in tonsil samples, CD56+ cells only displayed a statistically significant positive weak correlation with IFNγ+ cells (r = 0.268, *p* = 0.023, Spearman correlation test) in the entire cohort. When the EBV infection groups were analyzed separately, CD56+ cells showed a trend toward correlation with IFNγ+ cells in R patients (*p* = 0.079, r = 0.586, Spearman correlation test), whereas this correlation reached statistical significance in PI patients (r = 0.474, *p* = 0.030, Spearman correlation test). Furthermore, a statistically higher count of IFNγ+ cells was observed in PI compared to HC (*p* = 0.044, Mann–Whitney test) (Figure 3). CD56+ cells did not show a significant correlation with the Foxp3, CD68, IL10, TGFβ, and PDL1 markers (*p* > 0.05, Spearman correlation test) in both scenarios (Supplementary Figure S1).

Given the fact that EBV-associated lymphomas are significantly associated with patients younger than 10 years of age [13], and the median age in this cohort is 5 years, mean comparisons as well as correlations were assessed by clustering data into two types of analysis: younger versus older than 5 years, and younger versus older than 10 years of age, to disclose the effect of age on the IFNγ+ cell count. No differences in the IFNγ+ cell count were observed in children older and younger than 10 years (*p* > 0.05, Mann–Whitney test). However, only in children younger than 5 and 10 years a statistically significant positive correlation between CD56+ cells and IFNγ+ cells observed (r = 0.402, *p* = 0.018 for 5 years; r = 0.306, *p* = 0.017 for 10 years; Spearman correlation test).

The latency II profile (EBERs+, LMP1+, EBNA2−) is a defining feature of EBV-associated cHL [18]. Although the expression of viral antigens in tonsils is limited to a small number of cells, our group previously demonstrated the expression of latency antigens in the tonsils of children with asymptomatic infection, ranging from Latency I to Latency III antigens [15]. Therefore, patients who underwent tonsillectomy were clustered according to whether their tonsillar sections expressed latency 0–I and II–III antigens to evaluate the influence of latent EBV protein expression on the CD56 marker. Mean cell counts for CD56+ cells displayed no differences between cases expressing different latency antigens (*p* > 0.05, Mann–Whitney test). However, in children whose samples expressed latency 0/I antigens, a statistically significant positive correlation was observed between CD56+ cells and IFNγ+ cells (r = 0.431, *p* = 0.018, Spearman correlation test). Furthermore, EBNA2+ cells also exhibited a statistical positive correlation with IFNγ+ cells in PI children (r = 0.429, *p* = 0.046, Spearman correlation test), while the remaining correlations between viral proteins and CD56+ cells did not reach statistical significance (*p* > 0.05, Spearman correlation test). No differences were observed in CD56+ cells in the presence or absence of BMRF1 viral lytic antigens (*p* > 0.05, Mann–Whitney test).

### 3.3. IFNγ Production by NK Cells

**Figure 4:**
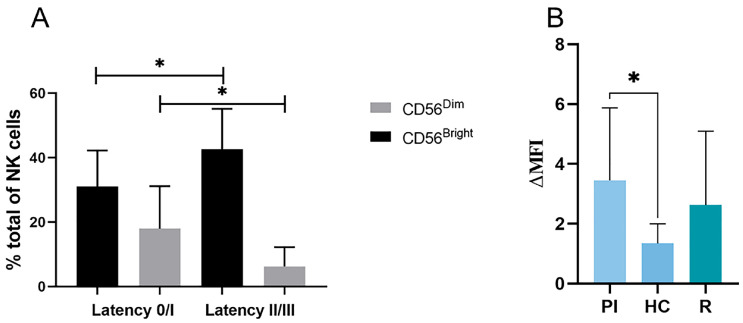
FC assay to assess NK subpopulations. (**A**) Comparison of CD56 bright and CD56 dim cells between latency 0/I and latency II/III patterns. (**B**) Comparison of IFNγ production (MFI normalized to the non-stimulated control and represented as ΔMFI) among PI, HC and R patients, gating on CD56bright NK cells. * *p* < 0.05.

It has been reported that the presence of a specific subpopulation of NK cells in the tonsils limits primary EBV infection until adaptive immunity establishes immune control, via secretion of IFNγ, which is also responsible for the control of the viral transforming capacity [19,20]. Given that a correlation with CD56+ cells was observed only with IFNγ, this cytokine production by NK cells was analyzed by FC in a subgroup of 27 patients (8 PI, 12 HC and 7 R) who underwent tonsillectomy. A sufficient number of NI patients to perform robust statistical analyses was not obtained. Initial phenotypic analysis showed that CD56bright cells were prevalent among tonsillar NK cells, as expected. No significant differences among the EBV infection groups were observed in the numbers of total NK cells (CD3-CD56+), CD56dim cells, or in the CD56bright population (*p* > 0.05, Kruskal- Wallis test). A higher mean number of CD56bright cells was found in those cases expressing latency II or III antigens when compared with cases showing latency 0 or I (*p* = 0.038, Mann–Whitney test). In contrast, a statistically lower mean number of CD56dim cells was observed in patients expressing latency II or III antigens (*p* = 0.046, Mann–Whitney test) (Figure 4A).

Given that CD56bright cells prevailed, their capacity to produce IFNγ upon stimulation with a rhIL-12/15/18 combination by CD56bright cells was analyzed. Gating on CD56bright NK cells, IFNγ production, compared to the unstimulated control, was statistically higher in the PI group when compared with HC patients (*p* = 0.0196, Kruskal–Wallis test, *p* = 0.0151 Dunn’s multiple comparisons test) (Figure 4B). In contrast, no differences in IFNγ production upon stimulation were observed concerning latency 0/I vs. II/III antigens expression (*p* > 0.05, Mann–Whitney test). Surprisingly, a statistically significant negative correlation was observed in EBV-infected children between IFNγ production by MFI and CD56bright cells (r = −0.470, *p* = 0.024; Spearman correlation test), and this correlation was significant in HC when EBV infection statuses were analyzed separately (r = −0.4697; *p* = 0.0237, Spearman correlation test).

## 4. Discussion

It has been previously reported that NK cells have a significant role in herpesvirus infections [21]. In the context of EBV infection, the tonsillar NK cell population can control the viral infection and inhibit its transforming capacity in vitro [19]. In cHL, soluble factors released by either tumor cells or infiltrating immune subpopulations can impair NK cell recruitment and activation [18]. The immunosuppressive cytokines IL10 and TGFβ, frequently abundant in the cHL milieu, reduce the lymphocyte production of IFNγ, thus impairing NK cell recruitment [22]. Even when NK populations resist the unfavorable cHL microenvironment, they are often excluded from the vicinity of Reed–Sternberg (HRS) cells [23]. Although the microenvironment composition was explored by several groups, including ours, in pediatric cHL [24,25,26], CD56 expression, as an indirect marker of NK cells, remains uncharacterized in both EBV+ and EBV− cases. In this cohort, despite the fact that cytotoxic CD8+ along with an M1 polarized microenvironment was demonstrated in EBV+ cHL cases [26], our findings using an immunohistochemical approach suggest that CD56+ cells, as an indirect marker of NK cells, are unlikely to be involved in the establishment of this virus-driven microenvironment, since no differences were observed between EBV+ and EBV− cases. CD56+ cells showed a significant positive correlation only with CD4+ T cells, particularly in EBV-associated pediatric cHL, and further studies are warranted to elucidate how CD4 T cells may interact with CD56+ cells in this context. The absence of a correlation between CD56+ cells and the remaining analyzed markers, Foxp3, CD68, IL10, TGFβ, and PDL1, suggests that CD56+ cells, as an indirect marker of NK cells, may not play a central role in modulating the recruitment or expression of these specific immunoregulatory components within the microenvironment. In the tonsils of pediatric patients who are EBV carriers, our group previously described that the CD56+ cell count around the EBV+ zones was lower than the count in the EBV− areas [13]. In adults with IM, also using a CD56 antibody to identify NK cells, CD56+ NK cells were the least prevalent cell subset in the tissue microenvironment, and tended to negatively correlate with EBERs+ B cells [12]. In children from a developed population with symptomatic IM, no differences in NK cell levels were observed between pediatric IM patients and HC [27]. In line with these findings, children in our cohort with primary asymptomatic infection showed no differences in CD56+ cells compared with HC, as studied by IHC.

In lymphoid organs, most NK cells are primarily involved in IFNγ production rather than cytotoxic activity [28]. In this series, as expected, CD56+ cells showed a statistically significant but weak positive correlation with IFNγ+ cells detected by IHC, which was more pronounced in PI patients. This finding may indicate the recruitment of IFNγ-producing CD56+ cells in response to primary EBV infection, although this remains an indirect marker so far. Such a response may be associated with a lower number of cells expressing latency II and III antigens, as suggested by the correlation between IFNγ+ and CD56+ cells detected by IHC in tonsillar samples expressing latency 0/I antigens. In addition, the significant correlation observed between EBNA2+ cells and IFNγ+ cells in tissue sections from PI children may reflect an increased contribution of IFNγ-producing cells other than CD56+ cells to the control of latency III antigen expression. The higher frequency of IFNγ+ cells detected by IHC in PI compared with HC further underscores the need for additional studies to better define the role of IFNγ in this context. Accordingly, IFNγ expression across distinct NK cell populations was assessed.

In order to further describe the immunohistochemical findings, FC was performed. This complementary approach allowed for a more detailed assessment of the indirect marker expression identified by IHC. It has been demonstrated that tonsillar CD56bright NK cells restrict primary EBV infection in B cells [29]. Moreover, IFNγ production was even higher when CD56bright cells were co-cultivated with dendritic cells [19]. In order to confirm whether CD56bright NK cells are directly responsible for the IFNγ production in PI children observed by IHC, and whether these cells are able to produce this cytokine, IFNγ production specifically in CD56bright cells was evaluated by FC. IFNγ production under stimulation was higher in PI children compared to HC, suggesting that this specific population may play a key role in primary EBV infection and, probably, in the lack of symptoms in these children. In contrast, the negative correlation between CD56bright cells and IFNγ producing cells may be particularly relevant in HC children. Therefore, in the context of persistent infection in HC, IFNγ production by this subset might not be a dominant mechanism for long-term viral control. Instead, alternative immune pathways or NK cell subsets may play a more relevant role in HC individuals. Our group previously reported the presence of CD8+PD-1+ T cells in EBV-infected children [30]; therefore, T cell exhaustion may represent a key mechanism underlying the impaired IFNγ-mediated response. Our FC observations confirm that the IFNγ+ cells detected by IHC are produced by CD56bright NK cells only in PI. In addition, our findings reveal the inefficiency of CD56bright cells in producing IFNγ during persistent infection in HC. Furthermore, the increased recruitment of CD56bright cells in patients who express latency II and III antigens may indicate unrestricted expression of latent antigens in this scenario.

This study has some limitations that should be acknowledged. First, the study is descriptive in nature, and the relatively small cohort size may limit the statistical power and generalizability of the findings. In addition, further in-depth functional studies are required to directly assess NK cell antiviral capacity and to better define their role in the control of EBV infection and persistence in pediatric populations.

## 5. Conclusions

In summary, this study provides novel insights into the role of NK cell response in a pediatric population characterized by a high incidence of early primary EBV infection, and EBV-associated pediatric lymphomas [14]. Our findings reveal that, despite the recruitment of CD56bright NK cells to tonsillar tissue, these cells appear to play a role only in PI children, rather than in HC with persistent EBV infection. However, this interpretation requires confirmation in other populations of children with asymptomatic infection, as well as further functional assays. Furthermore, the low number of CD56+ cells, as an indirect marker of NK cells, observed in pediatric HL may suggest that NK cells are unlikely to play a role in EBV-driven lymphomagenesis in children from our region.

## Data Availability

Data is contained within the article or Supplementary Material.

## Supplementary Materials

The following supporting information can be downloaded at: https://www.mdpi.com/article/10.3390/v18060667/s1, Figure S1: Correlation matrix of CD56+ cells with the analyzed markers by IHC in children undergoing tonsillectomy. Table S1: Infection status by EBV serological profile; Table S2: Characterization of HL patients.

## Abbreviations

The following abbreviations are used in this manuscript:

BL	Burkitt lymphoma
DLBCL	diffuse large B cell lymphoma
EA	early antigen
EBER	Epstein–Barr virus encoded RNA
EBNA	Epstein–Barr virus nuclear antigen
EBV	Epstein–Barr virus GzB: granzime B
HC	healthy carriers
HL	Hodgkin lymphoma
IHC	immunohistochemistry
IFN	interferon
IM	infectious mononucleosis
LMP	latent membrane protein
MFI	mean fluorescence intensity
NK	natural killer
PI	primary infection
R	reactivation
TMC	tonsillar mononuclear cells
TME	tumor microenvironment
