# Supplementary material for: Analysis of Tonsillar NK Cell Markers in Pediatric Epstein–Barr Virus (EBV) Asymptomatic Infection and EBV-Associated Hodgkin Lymphoma
Source: Viruses. 2026 Jun 12;18(6):667. doi: 10.3390/v18060667 (PMC13307723; doi:10.3390/v18060667)

Table S1. Infection status by EBV serological profile.

	VCA IgM	VCA IgG 1/10, 1/40, 1/320	EA IgG	EBNA 1 IgG	N
Primary infection	+	+/-	+/-	-	23
Healthy carrier	-	+	-	+	37
Reactivation	-/+	+	+	+	10
Not infected	-	-	-	-	4
Total					74

Table S2. Characterization of HL patients

	N	EBV status	
Histological Subtypes		EBV+	EBV-
Mixed cellularity	26	24	2
Nodular Sclerosis	10	6	4
Others (LR and LD)	3	0	3
Total	39	30	9

Abbreviations: LD, lymphocyte depletion; LR, lymphocyte rich.

Supplementary Figure S1: Correlation matrix of CD56+ cells with the analyzed markers by IHC in children undergoing tonsillectomy.

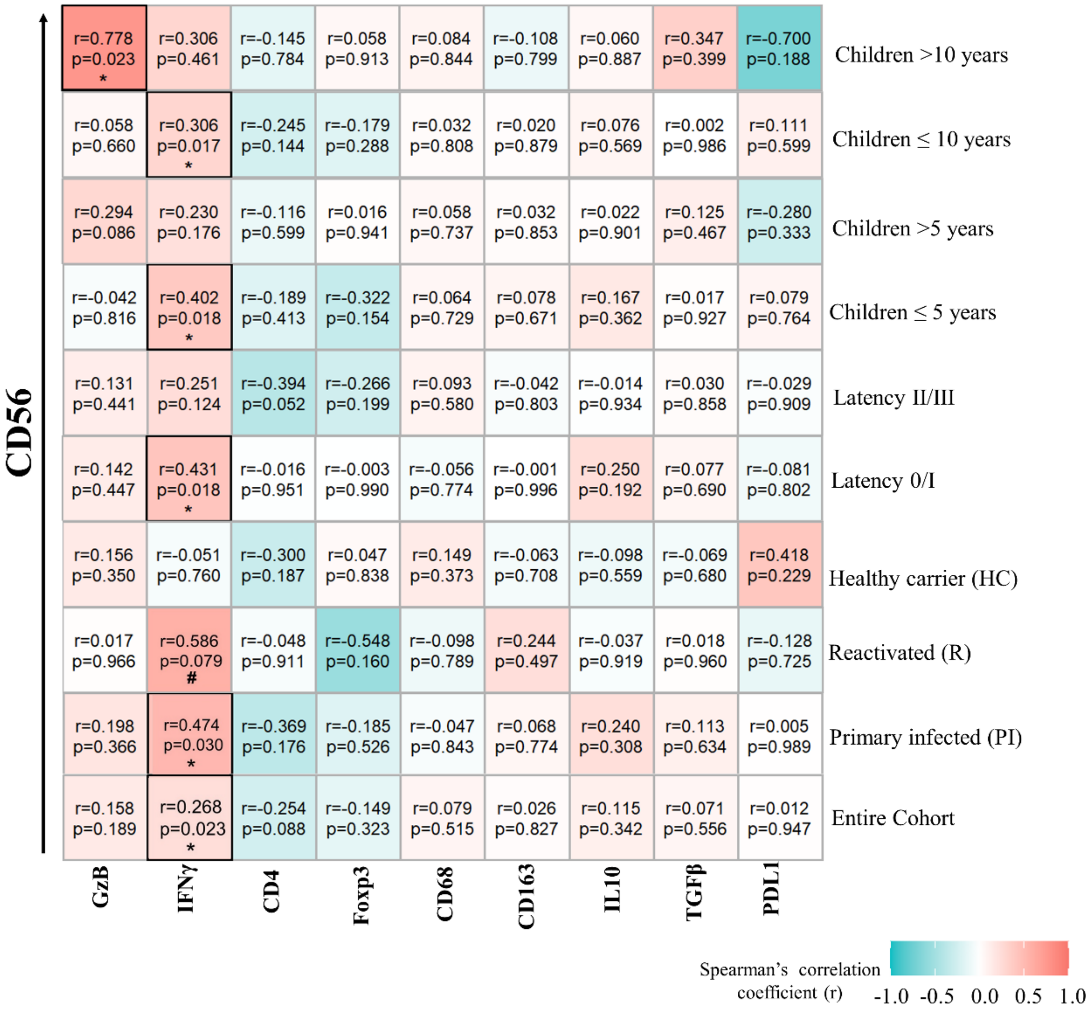

Supplement: Supplementary file 1 [file viruses-18-00667-s001.zip › viruses-4334297-supplementary.pdf]
